# Primary Care Quality among Different Health Care Structures in Tibet, China

**DOI:** 10.1155/2015/206709

**Published:** 2015-03-15

**Authors:** Wenhua Wang, Leiyu Shi, Aitian Yin, Zongfu Mao, Elizabeth Maitland, Stephen Nicholas, Xiaoyun Liu

**Affiliations:** ^1^School of Public Health, Wuhan University, 115 Donghu Road, Wuhan, Hubei 430071, China; ^2^Center for Health Management and Policy, Shandong University, No. 44 Wenhuaxilu, Jinan, Shandong 250012, China; ^3^Johns Hopkins Bloomberg School of Public Health, Johns Hopkins Primary Care Policy Center, 624 North Broadway, Baltimore, MD 21205, USA; ^4^School of Management, Australian School of Business, University of New South Wales, Sydney, NSW 2052, Australia; ^5^School of Management, Tianjin Normal University, West Bin Shui Avenue, Tianjin 300074, China; ^6^Guangdong Research Institute for International Strategies, Guangdong University of Foreign Studies, 2 Baiyun North Avenue, Baiyun, Guangzhou, Guangdong 510420, China; ^7^University of Newcastle, Newcastle, NSW 2308, Australia; ^8^China Center for Health Development Studies, Peking University, 38 Xue Yuan Road, Hai Dian District, Beijing 100191, China

## Abstract

*Objective.* To compare the primary care quality among different health care structures in Tibet, China. *Methods.* A self-administered questionnaire survey including Primary Care Assessment Tool-Tibetan version was used to obtain data from a total of 1386 patients aged over 18 years in the sampling sites in two prefectures in Tibet. Multivariate analysis was performed to assess the association between health care structures and primary care quality while controlling for sociodemographic and health care characteristics.* Results.* The services provided by township health centers were more often used by a poor, less educated, and healthy population. Compared with prefecture (77.42) and county hospitals (82.01), township health centers achieved highest total score of primary care quality (86.64). Factors that were positively and significantly associated with higher total assessment scores included not receiving inpatient service in the past year, less frequent health care visits, good self-rated health status, lower education level, and marital status.* Conclusions.* This study showed that township health centers patients reported better primary care quality than patients visiting prefecture and county hospitals. Government health reforms should pay more attention to THC capacity building in Tibet, especially in the area of human resource development.

## 1. Introduction

Considerable evidence has demonstrated the association between primary care quality and better health outcomes [1–9]. Primary care is the provision of first contact, comprehensive, accessible, and integrated health services for people within a geographical area. Good primary care quality promotes better population health status, lower health care costs, and the more equitable distribution of health within and across populations [1–9]. Starfield has identified structure, or the ability to achieve an attribute, and performance as the two key factors in assessing primary care quality [10]. Previous international comparative studies on primary care quality have revealed that different structures result in different health care outcomes and performance [10–14]. This paper compares Tibetan primary care quality across different health structures to assess outcomes and performance.

Health care outcomes and performance are also impacted by health care reforms, which influence the structure of primary health care provision. In 2009, China unveiled an ambitious health care reform program, encompassing the primary health care delivery system, as well as reforms in public hospitals, health insurance, the essential medicines system, and essential public health services. China's primary health care reforms involved increasing the per head funding of health care, differentials between rich and poor provinces in funding, tasking primary providers as health gatekeepers, and providing better training for doctors at primary health care facilities [15]. Some national reforms were specific to Tibet. For example, each township health center (THC) was funded by the national government to employ a minimum number of health staff, and an additional subsidy was allocated to village doctors (which will help to reduce health staff turnover). As an autonomous region, Tibet also had its own reform initiatives in addition to national reforms to the structure of its health care system. For example, the Tibet government expanded the Tibetan traditional medicine service, especially Tibetan medical departments in county hospitals and township health centers.

In Tibet, the health system is a primary care based system, comprising prefecture hospital (PH), county hospital (CH), and THC. Each health facility serves a large geographical area. For Tibet's 3.1 million people, the hierarchy of administrative arrangements comprises seven prefectures, ranging in size between 95 and 700 thousand people, 73 counties, and several towns. Each prefecture has one prefecture people's hospital, which mainly provides western medicine service, and one prefecture Tibetan medicine hospital, which mainly provides traditional Tibetan medicine services. There are on average 237 health staff in prefecture people's hospitals and 71 health staff in prefecture Tibetan medicine hospitals, with staff salary shared between the local hospital and national governments. In each county, there is one CH with on average 39.8 health staff, whose salary is fully paid by the national government. THCs play a more important role in Tibet than in other areas in China. The national government funds staff salaries, infrastructure cost, and equipment cost of THC, and part of the health reforms in Tibet involve the national government doubling the average number of 4.6 health staff in THC [16–18].

Patients can visit any health facility without restriction. Medical expenses in all of the three types of health facilities can be reimbursed through health insurance [16–18]. Similar to other areas of China, Tibet has three types of health insurance schemes to cover the whole population. In urban area, the Urban Employee Basic Medical Insurance (UEBMI) scheme is directly tied to formal employment status, and the voluntary household-based Urban Resident Basic Medical Insurance (URBMI) scheme aims to cover the rest of urban population. In rural areas, the New Cooperative Medical Scheme (NCMS) was developed to cover the rural population [19].

This paper addresses two major research questions: how is Tibet meeting the goal of improving the quality of primary health care services, and, second, how do different primary health care structures perform? Yip et al. noted that “no data have been reported for the quality or equitable distribution of public health services or primary health care services” [15]. This paper aims to address this lacuna. In the context of Tibet's rapidly changing health care system, our study assessed primary care quality across Tibet's three health care structures, prefecture hospitals, county hospitals, and township health centers, with the aim of providing information and advice for policy makers in Tibet.

## 2. Materials and Methods

### 2.1. Study Design

This study was based on face-to-face patient surveys conducted on-site at the sampled health care structures in Tibet. Based on socioeconomic and geographic factors, a stratified purposive sampling approach was used to select two prefectures, Shigatse and Linzhi. Tibet has seven prefectures, with each prefecture having several counties, each county including several townships, and each township including several villages. Comprising 18 counties, Shigatse prefecture has 630,000 residents with a per capita disposable income of 14700 RMB; and the share of economic activity equally balanced between agriculture (24 percent) and industry (25 percent). Linzhi is a smaller, less populated (173,000 residents) and more industrial (industry 35 percent and agriculture 15 percent of economic activity) prefecture than Shigatse. In Shigatse, two prefecture hospitals, two county hospitals, and four township health centers were selected, while in Linzhi, two prefecture hospitals, one county hospital, and two township health centers were chosen. The sample sizes were comparable to similar studies that showed for analysis a maximum sample size of 300 per group was needed for a significance level of 5% with a power of 90% [11–13]. Allowing that some collected questionnaires may contain missing data, 10 additional questionnaires were conducted at each township health center, 20 additional questionnaires at each county hospital, and 30 additional questionnaires at each prefecture hospital. Overall, the face-to-face patient surveys at the four prefecture hospitals (720 interviews), three county hospitals (360 interviews), and six township health centers (360 interviews) yielded 1440 interviews. The prefecture hospitals had a larger sample size in order to conduct within-group analysis.

Between September and October 2013, trained interviewers from the local health bureau conducted the face-to-face interviews with over 18-year-old patients, immediately after the patients had completed their health visit. Only patients who reported that the structure they visited was their regular setting for care were interviewed. Patients were given small gifts of appreciation (worth 10 RMB), upon completion of the interview. Most of patients approached agreed to take part in our interviews. While data could not be collected on those patients who refused to participate in the survey, the most common reason was the desire of the patients to travel immediately, sometimes involving long distances to their homes. Of the 1440 questionnaires administered, 54 questionnaires were deleted due to missing data, leaving 1386 completed questionnaires.

### 2.2. Measures

A validated Tibetan version of the Primary Care Assessment Tool (PCAT-T) was used for data collection. PCAT was developed by Johns Hopkins Primary Care Policy Center to measure the extent and quality of primary care services provided at a structure designated by patients as their main source of general care. Under different health care systems outside the United States, modified PCAT has shown good cross-cultural adaptability for assessing primary care quality attributes from the patient's viewpoint [20–24]. PCAT-T had been validated previously to ensure the survey had good validity and reliability for primary care assessment in Tibet [25].

Health care attributes were measured using PCAT-T's nine scales: first contact and continuity, comprehensiveness (medical care), comprehensiveness (social care), first contact (access), coordination, family centeredness, community orientation, same doctor, and stableness (see Appendix for details). A four-point Likert-type scale was applied to measure certainty as to whether a service was received or not, ranging from “1” (definitely not) to “4” (definitely). A neutral response of “not sure/do not remember” was provided for the lack of knowledge about a characteristic and for consistency with methods used in PCAT studies in other countries. The neutral response was assigned a median value of 2.5. We converted Likert scales to scores ranging from 25 to 100 by dividing the Likert scale by 4 and multiplying by 100. Means of item scores in the same scale yielded scale scores, and the primary care total score was the mean of nine scale scores [23].

### 2.3. Data Analysis

Our analysis compared the achievement of primary care quality attributes across Tibet's three structures of health care, comprising PHs, CHs, and THCs. First, chi-square tests were used to identify any differences in sociodemographic, health service use, and health status characteristics between the three structures. Then, an analysis of variance was performed to compare the separate and total primary care attributes between the three health care structures. Finally, multiple linear regression analysis was conducted to examine the association of health care setting with primary care attributes while controlling for sociodemographic, health service use, and health status characteristics.

## 3. Results

Patients' sociodemographics and health status differed significantly across the three health care structures, except for age and gender. As shown in Table 1, patients in THCs had the lowest education level (5.6% with junior high school and above) and the lowest income level (58.9% with annual household income below 30000 RMB), while patients from PH had both the highest education level (50.3% with a junior high school and above) and the highest income level (62.9% with annual household income above 30000 RMB). Patients from PHs had lowest proportion of self-rated healthy patients (79.3%). THCs had the highest proportion of patients having 4 times or more outpatient visit (55.3%) and the lowest inpatient rate (7%) (see Table 1).

Table 2 presents the comparative results of primary care quality among THCs, CHs, and PHs. Analysis of variance showed that THC patients had the highest primary care assessment total score (86.64) while patients in PHs had the lowest primary care assessment total score (77.42). There were no significant differences between the three structures on the scale of comprehensiveness (medical care). In all the 9 scales except for the same doctor and stableness scales, THC patients reported significantly higher scores on primary care assessments than PHs and CHs. PH patients reported the highest primary care assessment scores on the same doctor and stableness scales, but the lowest on all other scales. CHs ranked second on all item scales except on the stableness scale.

Controlling for sociodemographic, health care utilization, and health status characteristics, there was a significant association between health care structures and the primary care assessment total score. The average adjusted primary care score was nearly 5 points higher at CHs (*P* < 0.001) and more than 8 points higher at THCs (*P* < 0.001) than at PHs. Other factors significantly associated with higher primary care assessment total scores included not receiving inpatient service in the past year (*P* < 0.001), less frequent health care visits (*P* = 0.024), good self-rated health status (*P* = 0.024), lower education level (*P* = 0.012), and marital status (*P* = 0.046) (see Table 3).

## 4. Discussion

This is the first study to assess the quality of primary health care from the patients' perspective in Tibet during the implementation of China's 2009 health reform plan. Patients could visit any of Tibet's three health care structures, (i.e., THCs, CHs, and PHs) without restriction. However, patients' education, income, and health status differed significantly across the three health care structures. While patients visiting THCs had lower levels of education and lower income than patients visiting CHs and PHs, THCs had the highest proportion of patients self-rating as healthy. Besides income, limited geographic access was likely to be a major constraint on choice of health care facilities, since high travel costs relative to family income meant that patients located a long distance from a town tended to access THCs rather than CHs or PHs [26, 27]. Income and education level of CH and PH patients likely led them to have a higher health awareness and higher demand for perceived health care quality available in PHs and CHs. Further, high income patients were likely to be located closer to CHs or PHs or could afford the transportation cost to visit CH or PH for primary care, compared to poorer patients [28].

Importantly, patient assessment of Tibet's health care quality did not reflect the deeply entrenched preconceived view held by many patients that PHs and CHs were superior to THC. That is because the primary care quality measured in our study is an interpersonal quality assessed from the patients' perspective and not a measure of technical quality. Our data show that THCs had the highest primary care assessment total score, especially on the scales of first contact (access) and community orientation. The higher score for first contact (access) suggests that patients received health service without waiting for a long time and obtained their needed service more easily in THC than in PHs and CHs. The THCs' higher score on the community orientation scale can be explained by THCs' geographical proximity to patients relative to PHs and CHs. Residents in a town are relatively concentrated around their THC, where the THC health staff are more familiar with the health status of the local patients and with local-town health problems. However, THCs' lower score on the scales of same doctor and stableness can be explained by the low number of THC doctors, on average 4-5 health staff per THC; THC doctors need to fulfill other government required work in addition to providing health care services and a periodic staff rotation pattern in THCs. The Tibetan health reform to increase the minimum number of health care workers to 10 at each THC is appropriately targeted to address the low same doctor score at THCs. To increase stableness, health care reform needs to address the lack of medical equipment, and especially the skills to operate the equipment, at THCs. The lack of equipment and the skills to operate medical equipment meant some medical services could not be provided by THCs, which forced some patients to visit PHs and CHs, contributing to THCs' low stableness score [16–18].

Patients who did not receive inpatient care and had few hospital visits in the past year reported receiving higher primary care quality. That is because patients who have less health service utilization are more likely to be healthy and tend to give positive rating to their experience. The results of self-rated health also confirm this point [29]. Lower educated and married patients also reported a high primary care assessment total score. One of the explanations could be that lower educated patients are more likely to be friendly, more respectful, and less critical of health services; married patients have more social support, which might lead to positive rating of their experience [28].

There are several limitations in this study. First, a self-reported survey was used to assess patient experiences. While we cannot get technical quality information through patient survey approaches, self-report is the only way that people's actual experiences can be assessed. Second, this study only measures patients' experience of care rather than the outcome of primary care service. Further study is needed to examine how primary care attributes are related to actual health outcome. Such a study can help us identify the main primary care attributes, which are most closely related to outcomes so that limited resources can be used to focus on these areas.

## 5. Conclusion

Reforming China's health system, including the primary goal of improving primary health care, is a daunting task for China and offers lessons for other countries undertaking health care reform. This study showed that THC patients reported better primary care quality, except for the scales of same doctor and stableness, than patients using PHs and CHs. As an effective means of improving primary care services for local residents, government health reforms should pay more attention to THC capacity building in Tibet, especially in the area of human resource development.

## Figures and Tables

**Table 1:** Comparison of sociodemographic characteristics and healthcare services use among adult patients in different health care structures.

Characteristics	THC (%)	CH (%)	PH (%)	*P* value
	(*n* = 358)	(*n* = 336)	(*n* = 692)	
Sociodemographic characteristics				
Gender				0.051
Male	147 (41.1)	156 (46.4)	339 (49.0)	
Female	211 (58.9)	180 (53.6)	353 (51.0)	
Age				0.682
<60 years	316 (88.3)	301 (89.6)	607 (87.7)	
≥60 years	42 (11.7)	35 (10.4)	85 (12.3)	
Education				<0.001
Below junior high school	338 (94.4)	249 (74.1)	344 (49.7)	
Junior high school and above	20 (5.6)	87 (25.9)	348 (50.3)	
Occupation				<0.001
Employed	284 (79.3)	295 (87.8)	535 (77.3)	
Unemployed	74 (20.7)	41 (12.2)	157 (22.7)	
Income				<0.001
≤30000 RMB	211 (58.9)	167 (49.7)	257 (37.1)	
>30000 RMB	147 (41.1)	169 (50.3)	435 (62.9)	
Marital status				<0.001
Unmarried	54 (15.1)	60 (17.9)	206 (29.8)	
Married	304 (84.9)	276 (82.1)	486 (70.2)	
Health service utilization				
Number of PCP visits during the past year				<0.001
≤3	160 (44.7)	249 (74.1)	513 (74.1)	
≥4	198 (55.3)	87 (25.9)	179 (25.9)	
Whether inpatient during the past year				<0.001
Yes	25 (7.0)	124 (36.9)	142 (20.5)	
No	333 (93.0)	212 (63.1)	550 (79.5)	
Health status				
Self-rated health				0.003
Unhealthy	46 (12.8)	50 (14.9)	143 (20.7)	
Healthy	312 (87.2)	286 (85.1)	549 (79.3)	

THC = township health center; CH = county hospital; PH = prefecture hospital; PCP = primary care provider.

*P* value by chi-square test.

**Table 2:** Comparison of primary care assessment score among adult patients in different health care structures.

Characteristics	THC score mean (SE)	CH score mean (SE)	PH score mean (SE)	*P* value
First contact and continuity	95.40 (0.39)	90.93 (0.52)	85.23 (0.51)	<0.001
Comprehensiveness (medical care)	80.9 (0.99)	78.05 (1.03)	80.45 (0.67)	0.075
Comprehensiveness (social care)	90.33 (0.84)	85.39 (0.73)	81.91 (0.61)	<0.001
First contact (access)	80.16 (1.15)	69.21 (1.40)	60.04 (0.86)	<0.001
Coordination	91.65 (0.73)	84.39 (0.91)	77.65 (0.75)	<0.001
Family centeredness	90.34 (0.68)	87.93 (0.60)	84.18 (0.53)	<0.001
Community orientation	87.98 (0.87)	80.36 (0.85)	67.36 (0.78)	<0.001
Same doctor	66.42 (1.54)	70.95 (1.26)	74.44 (0.95)	<0.001
Stableness	45.37 (1.18)	43.14 (1.04)	48.75 (0.92)	0.001

Total	86.64 (0.49)	82.01 (0.50)	77.42 (0.38)	<0.001

Note: higher value indicates a more positive experience. Primary care scores are not adjusted for gender, age, income, education, occupation, marital status, whether inpatient during the past year, number of PCP visits, and health status.

THC = township health center; CH = county hospital; PH = prefecture hospital; SE = standard error.

*P* value by analysis of variance.

**Table 3:** Multivariate liner regression analysis on primary care assessment score.

Dependent variable: primary care achievement (total score)	*B* (95% CI)	SE	*P* value
Intercept	68.77 (63.32–74.22)	2.78	<0.001
Health care settings			
PH	—		
CH	4.66 (3.37–5.95)	0.66	<0.001
THC	8.49 (7.11–9.87)	0.70	<0.001
Health care service utilization			
Whether inpatient in the past year			
Yes	—		
No	2.65 (1.37–3.92)	0.65	<0.001
Number of PCP visits in the past year			
≤3	—		
≥4	−1.27 (−2.37–−0.17)	0.56	0.024
Health status			
Self-rated health			
Unhealthy	—		
Healthy	1.55 (0.20–2.89)	0.69	0.024
Sociodemographic characteristics			
Gender			
Male	—		
Female	−0.59 (−1.59–0.42)	0.51	0.252
Age			
<60 years	—		
≥60 years	1.00 (−0.62–2.62)	0.83	0.226
Income			
≤30000 RMB	—		
>30000 RMB	0.80 (−0.24–1.84)	0.53	0.132
Education			
Below junior high school	—		
Junior high school and above	−1.56 (−2.77–−0.35)	0.62	0.012
Marital status			
Unmarried	—		
Married	1.23 (0.02–2.44)	0.62	0.046
Occupation			
Employed	—		
Unemployed	1.17 (−0.14–2.47)	0.66	0.080

THC = township health center; CH = county hospital; PH = prefecture hospital; SE = standard error.

## Appendix

### Primary Care Assessment Tool-Tibetan Version

*First Contact and Continuity*

Do you have a regular checkup by the PCP before going somewhere else?
Do you see doctor for new health problem before going somewhere else?
Does your PCP see you the same day?
Does your PCP see you on the same day when you get sick and your PCP's clinic is closed?
Do you talk to the doctor/nurse who knows you best if you have questions?
Does your PCP give you enough time to talk?

*Comprehensiveness (Medical Care)*

Does your PCP give you advice about home safety, like getting air circulation or in storing medicine?
Do you have tests for your cholesterol level?
Do you have pressure consultation?
Do you have care for women's health or men's health and do regular checkup?

*Comprehensiveness (Social Care)*

Do you have a consultation about pressure at work and interpersonal conflicts?
Does your PCP give you advice about handling family conflicts?
Does your PCP give you advice about exercise?

*First Contact (Access)*

Is your waiting time >30 min?
Is it difficult for you to get medical care from your PCP when it is needed?

*Coordination*

Do you follow up with treatment and taking medicine?
Does your PCP talk with you about what happened at the visit and know the result of your visit?

*Family Centeredness*

When you need referral, does your PCP discuss different places, recommend a better place, and illustrate the reasons?
Does your PCP ask about your ideas about planning treatment for you or your family members?
Does your PCP introduce you and your family to the types of medicines you could possibly get and ask about your ideas before giving a prescription?
Does your PCP meet with members of your family if needed?
Would you recommend your PCP to a friend or relative?

*Community Orientation*

Does your PCP ever make home visits?
Does your PCP know about important health problems of your neighborhood?
Does your PCP make a survey of patients to see whether needs were met?
Does your PCP make a survey of community to find out health problems?

*Same Doctor*

Have you been taken care of by the same doctor/nurse in PCP?

*Stableness*

Would you change your PCP if it was easy to do so?

## References

[1] Starfield B. (1994). *Primary Care: Balancing Health Needs, Services, and Technology*.

[2] Shi L. (1992). The relationship between primary care and life chances. *Journal of Health Care for the Poor and Underserved*.

[3] Starfield B. (1994). Is primary care essential?. *The Lancet*.

[4] Shi L. (1994). Primary care, specialty care, and life chances. *International Journal of Health Services*.

[5] Bindman A. B., Grumbach K., Osmond D., Vranizan K., Stewart A. L. (1996). Primary care and receipt of preventive services. *Journal of General Internal Medicine*.

[6] Greenfield S., Rogers W., Mangotich M., Carney M. F., Tarlov A. R. (1995). Outcomes of patients with hypertension and non-insulin-dependent diabetes mellitus treated by different systems and specialties: results from the medical outcomes study. *Journal of the American Medical Association*.

[7] Forrest C. B. (2003). Primary care gate-keeping and referrals: effective filter or failed experiment?. *British Medical Journal*.

[8] Starfield B., Shi L., Macinko J. (2005). Contribution of primary care to health systems and health. *Milbank Quarterly*.

[9] Shi L., Starfield B. (2000). Primary care, income inequality, and self-rated health in the United States: a mixed-level analysis. *International Journal of Health Services*.

[10] Starfield B., Cassady C., Nanda J., Forrest C. B., Berk R. (1998). Consumer experiences and provider perceptions of the quality of primary care: implications for managed care. *Journal of Family Practice*.

[11] Wong S. Y. S., Kung K. K., Griffiths S. M. (2010). Comparison of primary care experiences among adults in general outpatient clinics and private general practice clinics in Hong Kong. *BMC Public Health*.

[12] Wang H. H. X., Wong S. Y. S., Wong M. C. S. (2013). Patients' experiences in different models of community health centers in southern China. *Annals of Family Medicine*.

[13] Shi L., Starfield B., Xu J., Politzer R., Regan J. (2003). Primary care quality: community health center and health maintenance organization. *Southern Medical Journal*.

[14] Sung N. J., Suh S. Y., Lee D. W. (2010). Patient's assessment of primary care of medical institutions in South Korea by structural type. *International Journal for Quality in Health Care*.

[15] Yip W. C.-M., Hsiao W. C., Chen W., Hu S., Ma J., Maynard A. (2012). Early appraisal of China's huge and complex health-care reforms. *The Lancet*.

[16] Center for Health Management and Policy (2010). *Linzhi Health Resource Survey Report*.

[17] Center for Health Management and Policy

[18] Center for Health Management and Policy (2010). *Ali Health Resource Survey Report*.

[19] Hu S., Tang S., Liu Y., Zhao Y., Escobar M.-L., de Ferranti D. (2008). Reform of how health care is paid for in China: challenges and opportunities. *The Lancet*.

[20] Shi L., Starfield B., Xu J. (2001). Validating the adult primary care assessment tool. *The Journal of Family Practice*.

[21] Pantoja T., Beltrán M., Moreno G. (2009). Patients' perspective in Chilean primary care: a questionnaire validation study. *International Journal for Quality in Health Care*.

[22] Pasarín M. I., Berra S., Rajmil L., Solans M., Borrell C., Starfield B. (2007). An instrument to evaluate primary health care from the population perspective. *Atencion Primaria*.

[23] Yang H., Shi L., Lebrun L. A., Zhou X., Liu J., Wang H. (2013). Development of the chinese primary care assessment tool: data quality and measurement properties. *International Journal for Quality in Health Care*.

[24] Lee J. H., Choi Y.-J., Sung N. J. (2009). Development of the Korean primary care assessment tool—measuring user experience: tests of data quality and measurement performance. *International Journal for Quality in Health Care*.

[25] Wang W., Shi L., Yin A., Lai Y., Maitland E., Nicholas S. (2014). Development and validation of the tibetan primary care assessment tool. *BioMed Research International*.

[26] Zhu L. (2005). Basic medical care service provision in rural area of Tibet. *Hunan Social Science*.

[27] Lai Y., Meng Q., Wang W. (2013). Efficiency analysis of medical health resource allocation in three prefectures of Tibet. *Chinese Health Economics*.

[28] Ross C. E., Wu C.-L. (1995). The links between education and health. *The American Sociological Review*.

[29] Pressman S. D., Cohen S. (2005). Does positive affect influence health?. *Psychological Bulletin*.

